# The Analysis of Pore Development and Formation of Surface Functional Groups in Bamboo-Based Activated Carbon during CO_2_ Activation

**DOI:** 10.3390/molecules26185641

**Published:** 2021-09-17

**Authors:** Krittamet Phothong, Chaiyot Tangsathitkulchai, Panuwat Lawtae

**Affiliations:** School of Chemical Engineering, Institute of Engineering, Suranaree University of Technology, Nakhon Ratchasima 30000, Thailand; pkrittamet@gmail.com (K.P.); pnwlawtae@gmail.com (P.L.)

**Keywords:** bamboo, activated carbon, physical activation, surface functional groups, pore development, carbonization temperature

## Abstract

Pore development and the formation of oxygen functional groups were studied for activated carbon prepared from bamboo (*Bambusa bambos*) using a two-step activation with CO_2_, as functions of carbonization temperature and activation conditions (time and temperature). Results show that activated carbon produced from bamboo contains mostly micropores in the pore size range of 0.65 to 1.4 nm. All porous properties of activated carbons increased with the increase in the activation temperature over the range from 850 to 950 °C, but decreased in the temperature range of 950 to 1000 °C, due principally to the merging of neighboring pores. The increase in the activation time also increased the porous properties linearly from 60 to 90 min, which then dropped from 90 to 120 min. It was found that the carbonization temperature played an important role in determining the number and distribution of active sites for CO_2_ gasification during the activation process. Empirical equations were proposed to conveniently predict all important porous properties of the prepared activated carbons in terms of carbonization temperature and activation conditions. Oxygen functional groups formed during the carbonization and activation steps of activated carbon synthesis and their contents were dependent on the preparation conditions employed. Using Boehm’s titration technique, only phenolic and carboxylic groups were detected for the acid functional groups in both the chars and activated carbons in varying amounts. Empirical correlations were also developed to estimate the total contents of the acid and basic groups in activated carbons in terms of the carbonization temperature, activation time and temperature.

## 1. Introduction

Activated carbon is one of the most widely used solid adsorbents for separation and purification processes in both gas [1] and liquid [2] systems. Activated carbon is an amorphous carbon-based material which exhibits a high degree of porosity, an extended surface area, microporous structure, high adsorption capacity and a high degree of surface reactivity [3]. Typically, activated carbon can be synthesized from a variety of carbonaceous materials including cherry stone [4], sugar cane bagasse [5], palm stone [6], agricultural waste [7], hemp stem hemicellulose [8], and bamboo [9,10] or coal [11], lignite [12], and peat [13]. Sewage sludge, the residual material produced as a by-product from sewage treatment plants and which contains large amounts of organic matter, could be another potential source for activated carbon production, since the biochar produced from the sewage sludge showed a reasonably high carbon content and surface area of 47% and 152 m^2^/g, respectively [14,15]. The elementary structures of activated carbon are graphene layers and quasi-graphitic fragments that are composed together, which are referred to as microcrystalline structures. The microcrystalline structures randomly connect together forming a porous activated carbon. Consequently, the disorganized structure of activated carbon produces certain unique properties for the adsorbent, e.g., high surface area and porosity, a wide range of surface functional groups and a distribution of pore sizes [16]. These characteristics increase the flexibility of activated carbon in adsorbing a wide variety of adsorbate molecules.

The pore structure of activated carbon is distinguished by the volume distribution of various pore sizes (micropores, mesopores and macropores). This pore size distribution and pore connectivity have a direct influence on the diffusion rate of an adsorbate to the adsorption sites, while the adsorption capacity for an adsorbate is determined by the specific surface area and pore volume of activated carbon. In addition to the pore structure effect on the adsorption kinetics and equilibrium, the nature of surface chemistry also affects the selectivity or specificity for a given adsorbate by activated carbon. Activated carbon contains heteroatoms, for example, oxygen, nitrogen, hydrogen, sulfur, etc. and these atoms can react with the oxidizing agent during the activation process, leading to the formation of various surface functionalities on the carbon surface.

Commercial activated carbon is often produced by a two-step physical activation method, consisting of a char preparation step by the carbonization of a precursor in an inert atmosphere at a mid-high temperature (400–600 °C) [3] and followed by a char activation step at a relatively high temperature (800–1100 °C) to increase the internal porosity by gasification with an oxidizing agent such as steam, CO_2_, H_2_O_2_, and O_2_ [17,18,19,20,21]. Porous properties and surface chemistry of the resulting activated carbon depend on the type of raw materials, the carbonization conditions (time and temperature) and activation conditions (time and temperature) [22,23,24]. It is of prime importance to have detailed knowledge, both quantitatively and qualitatively, concerning the effect of the aforementioned preparation conditions on the pore development and the formation of surface functional groups, which in turn will benefit the selection of the most suitable activated carbon to meet a specific application.

It is therefore the purpose of this work to produce activated carbon from a bamboo precursor by the conventional two-step activation process using carbon dioxide as the activating agent and the variables studied were carbonization temperature, activation time and activation temperature. Porous properties of the prepared activated carbon samples were measured and correlated mathematically with the preparation variables. The mechanism of pore development as a function of the extent of gasification reaction was proposed to gain a better understanding of the influence of the activated carbon preparation conditions. The type and amount of oxygen functional groups during char activation were also determined and appropriate empirical equations were proposed to correlate the concentration of the surface functional groups with the conditions of activated carbon synthesis.

## 2. Results and Discussion

### 2.1. Precursor Characterization

Table 1 shows the proximate and ultimate analyses of the precursor used in this work. The results indicate that bamboo wood has a high content of volatile matter (73.02%) and low composition of ash. The fixed carbon of bamboo is 19.91% which is comparable to other biomass materials, e.g., 18.27% for oil palm fiber [25], 19.08% for eucalyptus, 17.15% for wattle wood [26], 16.74% for palm kernel, 11.83% for cassava pulp [27], 19.6% for longan seed [28], and 26.6% for bamboo [29]. For the ultimate analysis, carbon and oxygen were the major elements since they are parts of the biomass structure which consists of cellulose, hemicellulose, and lignin.

**Figure 1:**
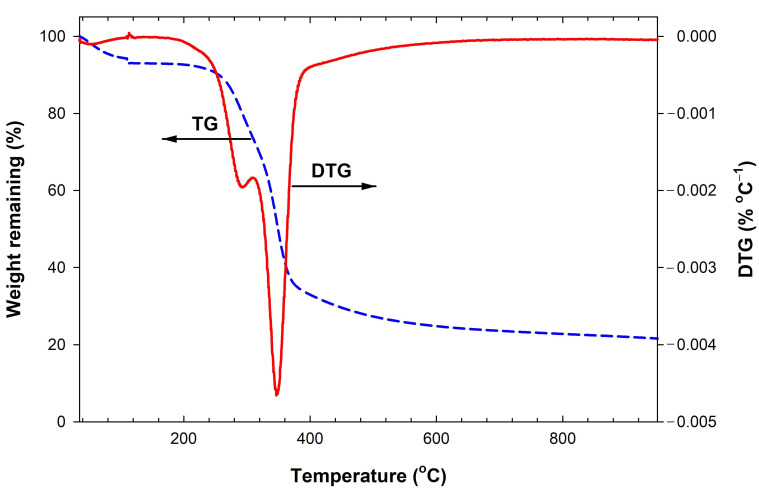
TG and DTG curves of bamboo biomass used in this work.

The thermogravimetric analysis was employed to investigate the thermal decomposition behavior of bamboo in a nitrogen atmosphere and the results are shown in Figure 1, for the TG and DTG curves. The TG curve shows the weight remaining versus the heating temperature, while the DTG curve gives the first derivative of the TG curve or the decomposition rate. The first detected small peak of the DTG curve at the temperature below 110 °C is attributed to the vaporization of residual moisture from the bamboo wood, which gives a weight loss of about 7%. Next, there are two observed peaks of the DTG curve that are related to the devolatilization process. The first peak occurs over the temperature range of 180 to 300 °C and the second peak from 300 to 390 °C. The maximum devolatilization rate of bamboo wood occurs at 345 °C for the second peak whereas the first small peak occurs at 290 °C. Lignin usually decomposes first at a low temperature around 150 °C and continues to decompose up to the temperature of 900 °C [30]. The first peak of the DTG curve from 180 to 300 °C should represent the decomposition of hemicellulose, since the range of the decomposition temperature agrees with that of the decomposition of the commercial hemicellulose [30]. Finally, the thermal decomposition of cellulose has been reported to occur over a higher temperature range from 315 to 400 °C [30]. Therefore, the appearance of the second DTG peak (300–390 °C) should be attributed to the decomposition of the cellulose component.

### 2.2. N_2_ Isotherms of the Prepared Activated Carbons

**Figure 2:**
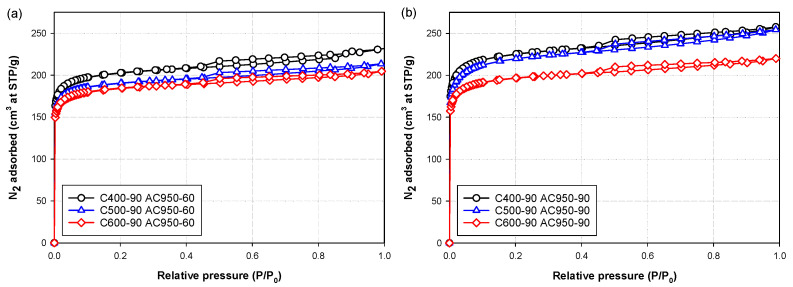
Effects of carbonization temperature and activation time on N_2_ adsorption isotherms of activated carbon prepared by CO_2_ activation at (**a**) 950 °C and 60 min (**b**) 950 °C and 90 min.

**Figure 3:**
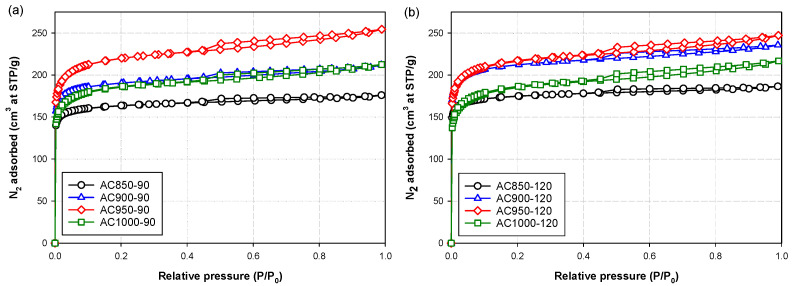
Effects of activation temperature and activation time on N_2_ adsorption isotherms of activated carbon prepared by CO_2_ activation at (**a**) 850 to 1000 °C and 90 min (**b**) 850 to 1000 °C and 120 min. The activated carbons were derived from char carbonized at 500 °C and 90 min.

**Figure 4:**
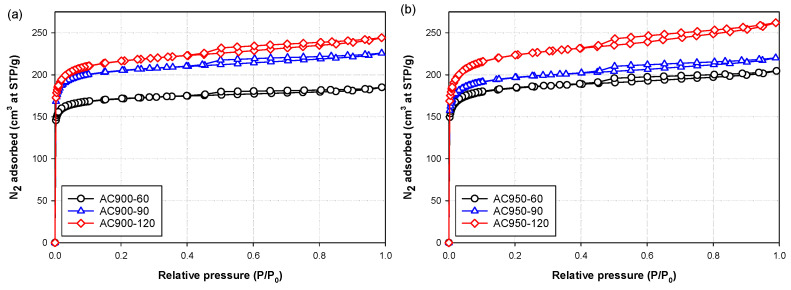
Effects of activation time on N_2_ adsorption isotherms of activated carbon prepared by CO_2_ activation at (**a**) 900 °C and (**b**) 950 °C for 60, 90 and 120 min. The activated carbons were derived from char carbonized at 600 °C and 90 min.

Effects of carbonization temperature, activation temperature and activation time on the nitrogen isotherms at −196 °C (77 K) of the prepared bamboo activated carbons are typically shown in Figure 2, Figure 3 and Figure 4, respectively. Obviously, the shape of the isotherm curves is dictated by the pore structure and pore size distribution of the adsorbent. The results indicate that two types of isotherm curves can be identified depending on the preparation conditions. They are Type I isotherm of the IUPAC classification [31] with a very small hysteresis loop and Type II isotherm [31] with a larger size of the hysteresis loop. Type I isotherm with a small hysteresis loop indicates the adsorption in micropores by a pore filling mechanism, followed by multilayer adsorption in small numbers of mesopores. This type of isotherm was found in activated carbon prepared at a relatively low activation temperature, as demonstrated in Figure 3 for samples AC850-90 and AC850-120. Type II isotherm with a larger hysteresis loop indicates the adsorption in micropores and adsorption in mesopores of proportionally larger volume, thus changing the isotherm shape from a flat plateau region for Type I isotherm to an isotherm with a linear increase in the adsorbed amount with an increase in the relative pressures. This type of isotherm is typified by activated carbon prepared at activation temperatures higher than 850 °C.

It is further observed from the adsorption isotherms in Figure 2 that the amount of N_2_ adsorbed tended to decrease for activated carbon prepared at a higher carbonization temperature. This behavior should be attributed to the difference in the char reactivity which affects the rate of CO_2_ gasification during the activation step, thus producing differences in the porous properties of the derived activated carbon. A detailed discussion on this aspect is presented in the next section.

The effect of activation temperature on N_2_ isotherms of activated carbon is displayed in Figure 3 at two activation times of 90 and 120 min, for activated carbon prepared from char carbonized at 500 °C for 90 min. It was observed that increasing the activation temperature from 850 to 950 °C increased the nitrogen isotherms which resulted from the increasing gasification rate that consumed more carbon atoms, thus providing an increase in the porous properties of the activated carbon. However, further increase in the activation temperature to 1000 °C gave rise to a decrease in N_2_ adsorption. This can possibly be ascribed to the reduction in the surface area of the activated carbon caused by either the coalescence of a number of adjacent pores arising at a high degree of char burn-off or the enlargement of the existing small pores at such a high activation temperature [32].

Figure 4a, b depict the effect of activation time on nitrogen isotherms for the activated carbon prepared from char carbonized at 600 °C for 90 min at the activation temperature of 900 and 950 °C, respectively. The amount of N_2_ adsorbed increased with the increase in activation time. Increasing activation time will allow more CO_2_ molecules to diffuse to different reaction sites and/or increase the probability of molecular collision for a successful gasification reaction.

### 2.3. Porous Properties of the Prepared Activated Carbons

**Figure 5:**
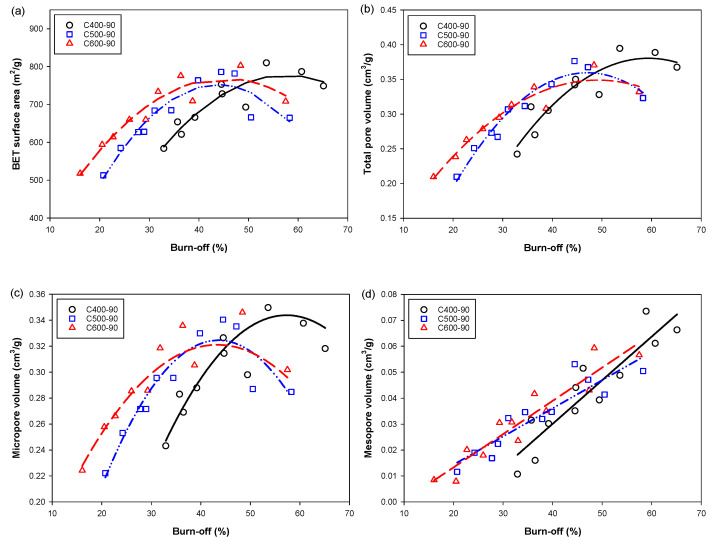
Effects of char burn-off on porous properties of the prepared activated carbons. (**a**) BET surface area, (**b**) total pore volume, (**c**) micropore volume, and (**d**) mesopore volume.

**Figure 6:**
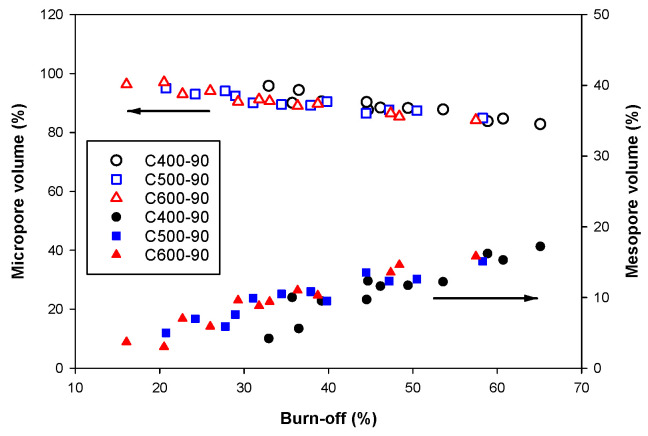
Effects of char burn-off on the percentage of micropore volume and mesopore volume. The blank symbols represent the micropore volume while the solid symbols represent the mesopore volume.

The effects of preparation conditions on the porous properties of the derived activated carbons are tabulated in Table A1 (see Appendix A). To ease the discussion, the results of porous properties of the activated carbon produced are presented graphically, as shown in Figure 5, to show the effects of char burn-off and the carbonization temperature. The char burn-off is a parameter that incorporates the effects of activation time and temperature of the activation step. It was observed that the BET surface area, the micropore volume and the total pore volume appeared to increase with the increase in char burn-off and passed through a maximum at a critical char burn-off. The decrease in these porous properties is hypothesized to result from either the enlargement of the existing pores or the consolidation of neighboring pores, or possibly from both mechanisms. If these hypotheses are plausible, it indicates that the reactivity and the distribution of the reaction sites on the carbon surfaces for the gasification reaction could exert a considerable influence on the pore development of the resulting activated carbon. On the other hand, there was a tendency for the mesopore volume to increase continuously with the increase in char burn-off. However, the maximum percentage of mesopore volume for the maximum char burn-off of 65% is only 20% of the total pore volume, as shown in Figure 6. Results from Table A1 also indicate that the maximum char burn-off is only about 65%, even the chars were activated at a very high activation temperature (1000 °C) and with a long activation time (120 min). This is possibly attributable to the dense and tenacious cellulosic structure of the bamboo which causes the production of activated carbon from bamboo to contain mostly micropores.

It is interesting to note from Table A1 that the maximum surface area of 907 m^2^/g was obtained for bamboo-activated carbon under moderate preparation conditions for char carbonized at 400 °C and 90 min and activation conditions of 900 °C and 120 min with about 46.2% char burn-off for the two-step activation in CO_2_. Apart from the conventional two-step physical activation (carbonization/activation), the one-step gasification in an oxidizing gas by heating a precursor from room temperature to the desired activation temperature can also be utilized for the production of activated carbon. As an example, Liu et al. [33] synthesized activated char briquette from the pine sawdust briquette at the maximum gasification temperature of 800 °C under 100% CO_2_ using the one-step heating that produced the activated biochar with reasonable BET surface area of around 478 m^2^/g. However, the application of the two-step activation is more advantageous for the reason that it could effectively remove tarry materials during the first carbonization step, giving better quality char for the following activation step.

**Figure 7:**
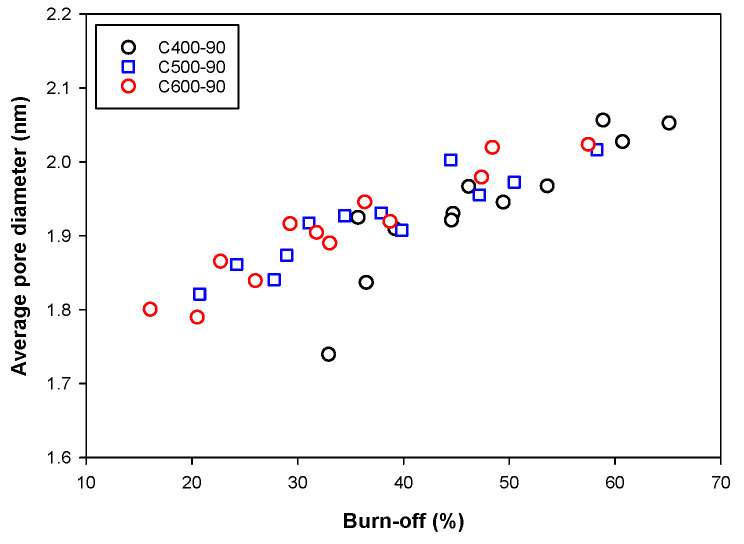
Effects of char burn-off on the average pore diameter of the prepared activated carbons.

Next, Figure 5 shows that for up to 50% of char burn-off, the porous properties of activated carbon increased with the increase in carbonization temperature. This is the result of char being carbonized at a higher temperature having a higher initial surface area (see Table 2). Therefore, for a given char burn-off, the new area formed by gasification for each char must be equal, thus making the char with a higher initial surface area (higher carbonization temperature) possess a higher total surface area after the activation process. Figure 7 shows the effect of char burn-off on the average pore diameter (*D_avg_*) of activated carbon. The average pore diameter varied over a narrow range with the value increasing roughly in the range from 1.74 to 2.05 nm when the char burn-off increased from 16.9 to 65.1%, and the carbonization temperature appeared to have no significant effect on the average pore size.

**Figure 8:**
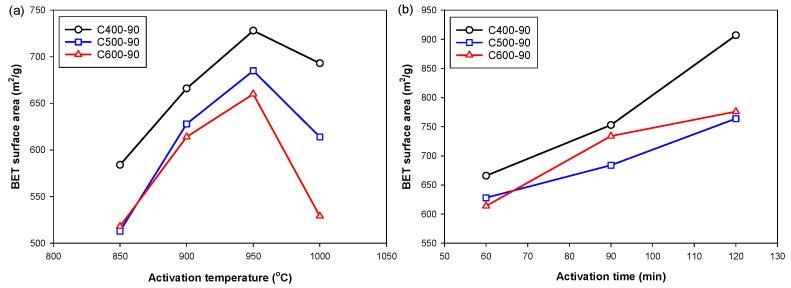
Effects of activation temperature and activation time on BET surface area of activated carbon prepared from chars carbonized at different temperatures (400, 500, and 600 °C) for (**a**) activation temperatures were varied from 850 to 1000 °C while the activation time was kept constant at 60 min and (**b**) activation times were varied from 60 to 120 min while the activation temperature was kept constant at 900 °C.

Figure 8 shows the effects of activation temperature and time on one of the important porous properties of activated carbon, the BET surface area, as a function of carbonization temperature. For activated carbon prepared from each carbonization temperature in the range from 400 to 600 °C, the specific surface area increased approximately linearly over the increase in activation temperature from 850 to 950 °C, but showed a rapid decrease from 950 to 1000 °C. The increase in the surface area is due mainly to the increase in the gasification rate with the increase in the reaction temperature, while the decrease in surface area above 950 °C is possibly the result of pore enlargement. This instance could occur when a considerable amount of carbon is consumed by the reaction, giving an increase in the average pore size of activated carbon and hence a consequent decrease in the surface area. Figure 8b shows that the increase in the activation time increased the activated carbon-specific area in an almost linear fashion. Increasing the activation time would increase the frequency of molecular collision for a successful reaction to occur or allow more time for CO_2_ to diffuse to the reaction sites, hence more carbon atoms in the graphene layers may have been removed for pore development.

**Figure 9:**
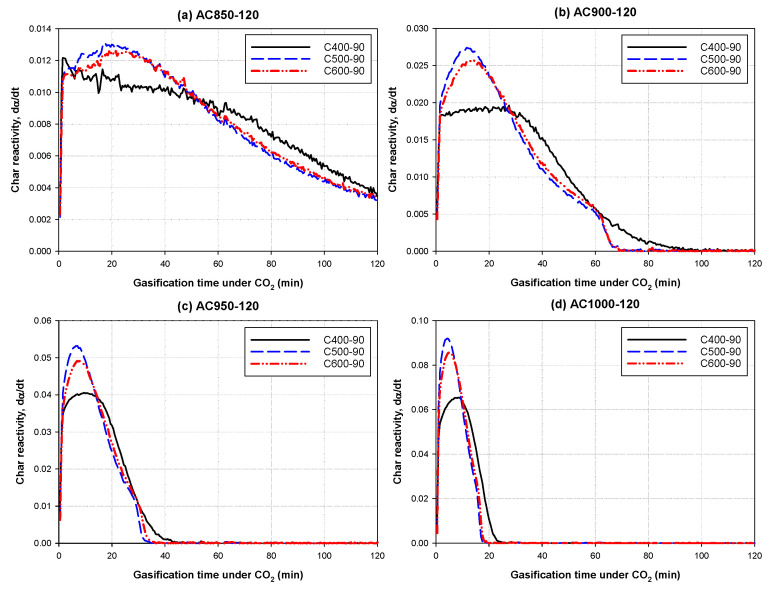
Variation of char reactivity during CO_2_ gasification as a function of time for various chars and activation temperatures. Lines with black, blue, and red colors represent chars prepared at 400, 500, and 600 °C, respectively, with each char being gasified at (**a**) 850 °C, (**b**) 900 °C, (**c**) 950 °C, and (**d**) 1000 °C for 120 min.

As to the effect of carbonization temperature, it is obvious that the surface area decreased with the increase in the carbonization temperature in the range of 400 to 600 °C, although the specific area of char prior to activation increases with increasing carbonization temperature (see Table 2). This signifies that activated carbon prepared at the lowest carbonization of 400 °C might be most reactive for CO_2_ gasification. To prove this hypothesis, bamboo chars prepared at 400, 500 and 600 °C in the tube furnace were subjected to CO_2_ gasification in a thermogravimetric analyzer (TGA/DSC-1 Star System, Mettler-Toledo, Greifensee, Switzerland) under conditions simulating the actual activation conditions. The char reactivity was defined here as the rate of fractional weight loss according to Equation (1) and were computed from the TGA data and plotted with respect to the gasification time, as shown in Figure 9.

The char reactivity for CO_2_ gasification (*R_c_*) can be defined as

(1) $$R_{c}=d\alpha /dt$$

where the fractional conversion of char, $\alpha =\left(w_{0}-w_{t}\right)/\left(w_{0}-w_{\infty }\right)$, and *w*_0_, *w_t_*, and *w*_∞_ are the weights of initial char, activated carbon at time *t* and the remaining ash, respectively.

Figure 9 shows the effect of gasification time on the values of char reactivity as functions of activation temperature (850 to 1000 °C) and carbonization temperature. The reactivity curves showed a characteristic rising and falling with reference to the increase in reaction time and demonstrated that the reactivity of char carbonized at the lowest temperature of 400 °C gave the lowest reactivity, but the char carbonized at the intermediate temperature of 500 °C produced the highest reactivity. This evidence suggests that although the char prepared at 400 °C (sample C400-90) had the lowest reactivity for the gasification, it could have had more reactive sites, which enabled the increase in the reaction rate and hence producing a larger developed surface area. Therefore, it is likely that carbonization temperature could have a profound effect on the number and distribution of reactive sites available during the activation step, which will control the resulting porous structure of the prepared activated carbon. This emphasizes the significance of carbonization temperature in determining the reactivity of char for the gasification reaction and the subsequent pore development in activated carbon.

**Figure 10:**
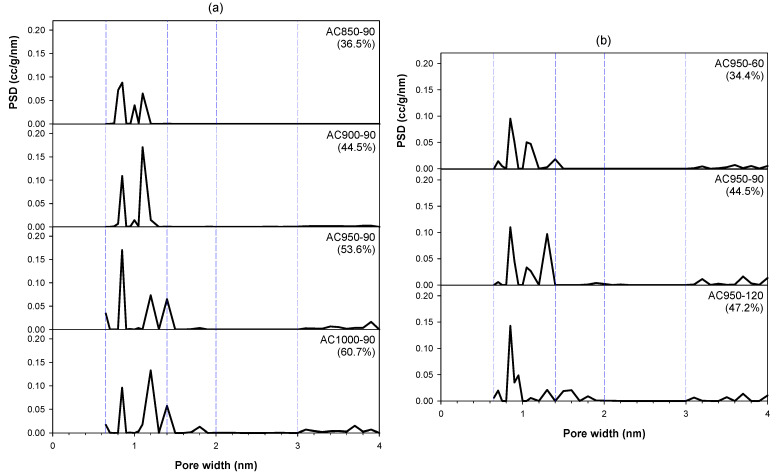
Effects of activation temperature and activation time on the pore size distribution of produced activated carbon for (**a**) char C400-90 and activation temperature of 90 min and (**b**) for char C500-90 and activation temperature of 950 °C.

We now turn to the effect of the preparation conditions on the pore size distribution of the produced activated carbon, which was computed based on the GCMC simulation procedure. Table 3 summarizes the distribution of pore sizes for all the prepared activated carbons, and the data are exemplified in Figure 10 to illustrate the effects of the activation conditions on the distribution of pore sizes. The pores developed in bamboo-based activated carbon show the characteristics of multimodal size distribution, which covers the micropore size range from 0.65 to 2.0 nm and mesopores from 2.0 to 4.0 nm. Most of the pores produced (>80% of total pore volume) were concentrated in the micropore size range of 0.65 to 1.4 nm. Some super-micropores (1.4–2.0 nm) were observed but no ultra-micropores (<1.4 nm) were detected in any of the activated carbons. Table 3 also shows that the mesopores were produced only in the size range of 3.0 to 4.0 nm with much smaller proportions as compared to the volume of the generated micropores. Figure 10 further shows that the increase in activation temperature from 850 to 950 °C increased the volume of the micropores in the size range from 0.65 to 1.4 nm, but the volume decreased slightly at the activation temperature of 1000 °C, corresponding to the creation of more mesopores. Again, this might explain the creation of mesopores at the expense of micropores at a high activation temperature by the mechanism of pore enlargement, as previously outlined.

The effect of activation time on the pore size distribution was similar to that of the activation temperature. The increase in activation time from 60 to 90 min increased the micropore volume but again it declined slightly at the longest time of 120 min, concomitant with the increase in the mesopore volume. Overall, it can be deduced that the number and distribution of reactive sites available for the CO_2_ gasification of the carbonized chars play a significant role in the formation of different pore sizes as well as the pore connectivity during the activation process for the synthesis of activated carbon from bamboo biomass.

### 2.4. Correlations for Porous Properties of the Prepared Activated Carbons

In general, the fluid-solid reaction models can be classified into two schemes: (1) the reaction taking place on the surfaces of nonporous grains, and (2) the reaction that occurs on the pore surface within the solid [34,35]. Of these models, the Random Pore Model (RPM) developed by Bhatia and Perlmutter [35] has been widely used in describing the gasification kinetics for porous solids, since it takes into account the effects of pore creation and the coalescence of neighboring pores at a high degree of char burn-off, thus showing a consequent decrease in the porous properties. Therefore, this model was adopted in this study to predict the development of porous properties of the obtained activated carbon from bamboo biomass. The final form of the random pore model (RPM) in terms of the developed surface area of solid can be written as:

(2) $$\frac{S}{S_{0}}=\frac{1-X}{{\left(1-\frac{\tau }{\sigma }\right)}^{3}}\sqrt{1-\psi \ln\left[\frac{1-X}{{\left(1-\frac{\tau }{\sigma }\right)}^{3}}\right]}$$

where *S* is the reaction surface area per unit mass, *S_0_* is the initial surface area per unit mass (the specific area of original char), *X* is the fractional conversion, *σ* is the particle size parameter, *τ* is the dimensionless time and *ψ* is the structural parameter. From Equation (2), it is convenient to assume σ→∞ since the particle external surface area can be negligible in comparison with the magnitude of the internal surface area. This gives:

(3) $$\frac{S}{S_{0}}=\left(1-X\right)\sqrt{1-\psi \ln\left(1-X\right)}$$

The structural parameter (*ψ*) was first calculated as a function of conversion based on Equation (3) using the surface area data of activated carbon (*S*), as shown in Table A1 and *S*_0_ for the surface area of the starting char which was estimated from CO_2_ isotherm data at 0 °C, as shown in Table 2. It is obvious that the structural parameter should depend on the conversion (*X*) because of the consequent change in the pore structure of the solid with the progression of reaction. It should be noted that the effect of activation time and temperature on the surface area (*S*) is accounted for by the char conversion (*X*). The relationship between the structural parameter and the char conversion can be approximated by the following power-law equation,

(4) $$\psi =C_{1}X^{C_{2}}$$

**Figure 11:**
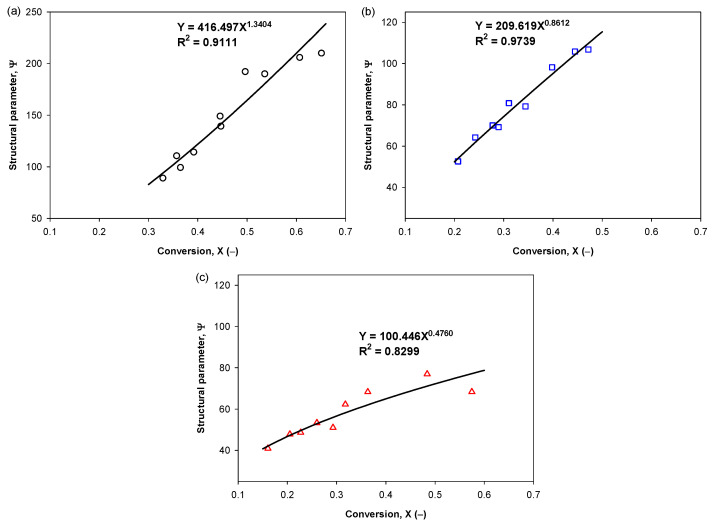
The relationship between the structural parameter and fractional conversion of char. Figure (**a**–**c**) refer to the activated carbon derived from char carbonized at 400, 500, and 600 °C for 90 min, respectively.

The fitting of the structural parameter as a function of char conversion is illustrated in Figure 11.

**Figure 12:**
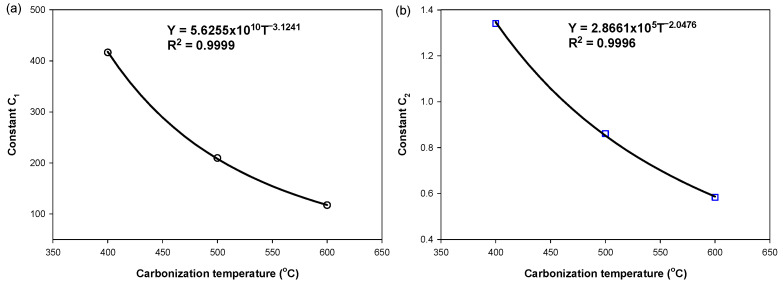
Correlations between (**a**) the constant *C*_1_ and (**b**) the constant *C*_2_ in Equations (5) and (6) and the carbonization temperature.

Next, the constants *C*_1_ and *C*_2_ were correlated with the carbonization temperature (*T_c_*) according to the power-law equation, as shown in the following equations,

(5) $$C_{1}=C_{3}T_{C}^{C_{4}}$$

(6) $$C_{2}=C_{5}T_{C}^{C_{6}}$$

and Figure 12 shows the fitting results.

Combining Equations (3)–(6) finally gives

(7) $$\frac{S}{S_{0}}=\left(1-X\right)\sqrt{1-C_{1}T_{C}^{C_{2}}X^{C_{3}T_{C}^{C_{4}}}\ln\left(1-X\right)}$$

Equation (7) was used to fit the measured surface area (*S/S*_0_) and the constants in the equation (*C*_1_, *C*_2_, *C*_3_, and *C*_4_) were evaluated by the non-linear regression. The final derived equation reads

(8) $$\frac{S}{S_{0}}=\left(1-X\right)\sqrt{1-5.6255\times 10^{10}T_{C}^{-3.1241}X^{2.8661\times 10^{5}T_{C}^{-2.0476}}\ln\left(1-X\right)}$$

**Figure 13:**
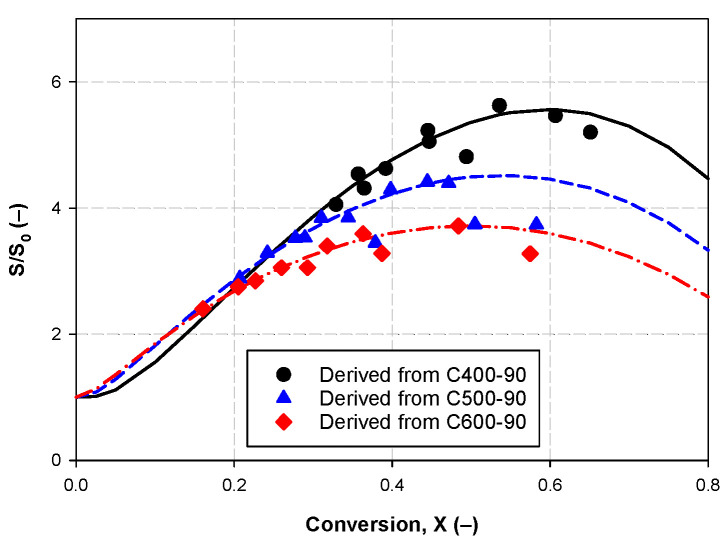
The development of reaction surface with conversion according to the RPM. The activated carbon products were derived from chars carbonized at 400, 500 and 600 °C for 90 min, respectively. The black solid line, blue dash line and red dash-dot lines represent the predicted data derived from Equation (8). The black circles, blue triangles and red diamonds indicate the experimental data points.

The coefficient of determination, R^2^, derived from fitting Equation (8) to the experimental data is 0.9788 with the standard error of 0.1826. Figure 13 compares the experimental and computed surface area results at three carbonization temperatures. Overall, the prediction capability of the random pore model (RPM) is satisfactory up to the maximum char conversion of about 0.65.

It appeared that there was an optimum char conversion or char burn-off that produced activated carbon with a maximum surface area and this optimum conversion depended on the carbonization temperature, being 0.597, 0.539 and 0.499 for the carbonization temperature of 400, 500 and 600 °C, respectively. The difference in the optimum char conversion with the change in the carbonization temperature should result from the difference in the char reactivity for the CO_2_ gasification, caused by the decomposition of cellulosic components of the precursor at different carbonization temperatures. The decrease in surface area at the conversion larger than the optimum one is possibly due to the association of adjacent pores into larger size pores. The fractional char conversion (*X*) is related to the activation conditions (time and temperature) by the following proposed equation.

(9) $$\begin{array}{l} X=-3.9708\times 10^{-1}-5.131\times 10^{-3}T_{C}+4.1554\times 10^{-3}T_{Act}-1.2122\times 10^{-2}t_{Act}+4.63\times 10^{-7}T_{C}T_{Act}+5.7333\times 10^{-6}T_{C}t_{Act}\\ \;+1.5213\times 10^{-5}T_{Act}t_{Act}+3.5213\times 10^{-6}T_{C}^{2}-2.2256\times 10^{-6}T_{Act}^{2}-1.4583\times 10^{-5}t_{Act}^{2} \end{array}$$

where *X* is the fractional conversion of char, *T_C_* is the carbonization temperature in °C, *T_Act_* is the activation temperature in °C, and *t_Act_* is the activation time in minutes, with R^2^ and standard error of estimate being 0.9923 and 0.0122, respectively.

It should be noted that Equations (8) and (9) can be used to estimate the surface area of activated carbon from bamboo precursor, knowing the carbonization temperature, activation temperature and activation time in the range of 400 to 600 °C, 850 to 1000 °C and 60 to 120 min, respectively.

Next, an attempt was made to develop an equation for estimating the total pore volume of activated carbon as a function of preparation conditions. It was found that the total pore volume (*V_tot_*) in cm^3^/g correlates approximately well with the BET surface area of activated carbon, according to the following power-law model:

(10) $$V_{tot}=aS_{BET}^{b}$$

where *a* and *b* are the constants of the equation and *S_BET_* is the BET surface area in m^2^/g of the obtained activated carbon. After fitting Equation (10) with the experimental data, the following final equation is derived.

(11) $$V_{tot}=1.314\times 10^{-4}S_{BET}^{1.199}$$

**Figure 14:**
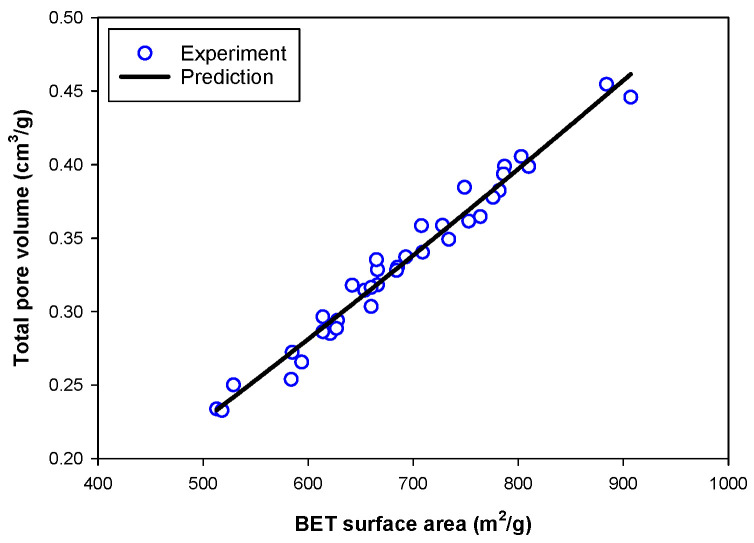
The correlation between total pore volume (*V_tot_*) and BET surface area. The black solid line is predicted from Equation (11), while the blue circles are the experimental data.

Figure 14 shows the agreement between the total pore volume determined from the N_2_ adsorption isotherms and that predicted by Equation (11), as a function of BET surface area. The coefficient of determination, R^2^, is 0.9738. This correlation can be employed to determine the total pore volume of the activated carbon from the corresponding BET surface area. The limitation of this correlation is that it can be used to predict the total pore volume in activated carbon obtained from bamboo precursor only and under the synthesis conditions used in the study.

Further work was devoted to the development of an empirical equation to correlate the micropore volume with the preparation conditions for activated carbon production. The following final equation was proposed.

(12) $$\begin{array}{l} V_{mic}=-8.5823-7.5308\times 10^{-4}T_{C}+1.9081\times 10^{-2}T_{Act}+3.5284\times 10^{-3}t_{Act}-1.3147\times 10^{-6}T_{C}T_{Act}+6.4792\times 10^{-7}T_{C}t_{Act}\\ \;-1.6811\times 10^{-6}T_{Act}t_{Act}+1.7525\times 10^{-6}T_{C}^{2}-9.7544\times 10^{-6}T_{Act}^{2}-7.4861\times 10^{-6}t_{Act}^{2} \end{array}$$

where *V_mic_* is the volume of micropore of activated carbon in cm^3^/g, *T_C_* is the carbonization temperature in °C, *T_Act_* is the activation temperature in °C, and *t_Act_* is the activation time in min. The R^2^ is 0.9241 with the standard error of 0.0134.

Finally, the equation for estimating the amount of mesopores volume (*V_mes_*) can be obtained from the difference between the total pore volume (*V_tot_*) and the micropore volume (*V_mic_*). That is,

(13) $$V_{mes}\left({cm}^{3}/g\right)=V_{tot}\left({cm}^{3}/g\right)-V_{mic}\left({cm}^{3}/g\right)$$

where *V_tot_* and *V_mic_* are computed from Equations (11) and (12), respectively.

In conclusion, all the important porous properties of activated carbon produced from bamboo by the two-step activation with CO_2_, including BET surface area, the total pore volume, the micropore volume, and the mesopore volume can be readily estimated from the developed equations as functions of the carbonization temperature (400 to 600 °C), activation temperature (850 to 1000 °C), and activation time (60 to 120 min).

### 2.5. Surface Chemistry of the Prepared Activated Carbons

**Figure 15:**
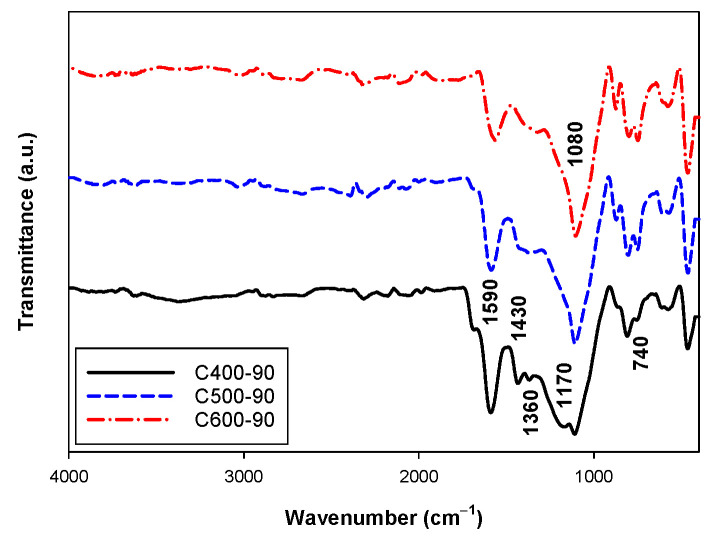
FTIR spectra of the prepared char. The black solid line, blue dash line, and red dash-dot line refer to char carbonized at 400, 500, and 600 °C for 90 min.

Fourier Transform Infrared Spectroscopy (FTIR) and elemental analysis were employed to analyze the development of surface functionality on the surface of activated carbon during the carbonization and the activation steps. First, three samples of char were selected for the analysis, which are char carbonized at 400, 500 and 600 °C for 90 min and the FTIR results are shown in Figure 15. The aromatic rings are characterized by the intense band at about 1590 cm^−1^; this is assigned to rings vibration in large condensed aromatic carbon [36]. This band is typically found in carbonaceous materials [37,38]. The small band at about 1360 cm^−1^ for the C400-90 sample corresponds to the existence of CH_3_ groups (CH-bending vibration) [38]. The weak band located at about 1170 cm^−1^ can be assigned to the C-O stretching vibration of phenol and aromatic ring of ether [29,39]. The broad band between 1220 to 1430 cm^−1^ of the C400-90 sample is attributed to nitrate, carboxyl and carboxylate groups [40]. The strong band at 1080 cm^−1^ was observed for all char samples, and this band is assigned to C-O stretching vibration of primary alcohols [40]. Lastly, the C-H out-of-plane bend at 740 cm^−1^ indicates the presence of aromatic ring in activated carbon [41].

The presence of the surface chemistry in the carbonized chars indicates that the different surface functional groups can be created during the carbonization step of the bamboo precursor. Next, we explore the surface chemistry of the activated carbon derived from the prepared char.

**Figure 16:**
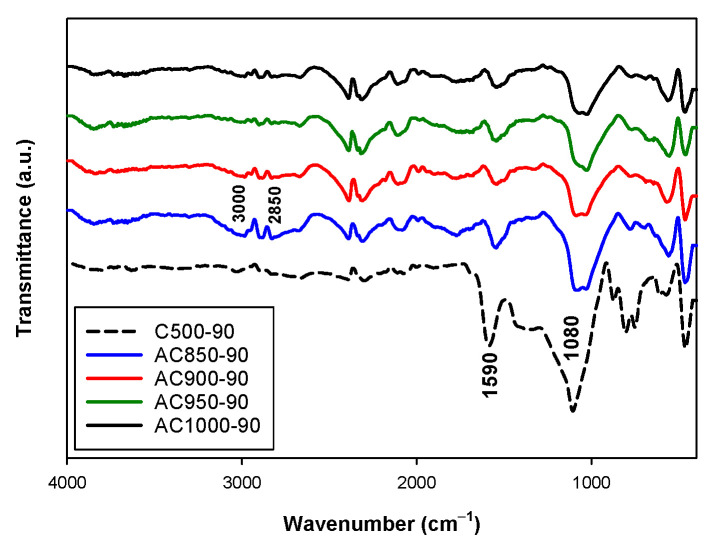
FTIR spectra of the obtained activated carbon. The blue, red, green, and black solid lines refer to activated carbon prepared at 850, 900, 950 and 1000 °C for 90 min. The black dash line is the char carbonized at 500 °C. All carbon samples were derived from char carbonized at 500 °C for 90 min.

FTIR spectra of activated carbon prepared at 850, 900, 950 and 1000 °C with the activation time of 90 min are typically shown in Figure 16. Here, we use char carbonized at 500 °C for 90 min as the starting char. The intense adsorption band at 1080 cm^−1^ was observed in the char sample, while the intensity of this band decreased for the activated carbon samples. This implies a decrease in C-O of primary alcohol structure. The weak band at about 1560 cm^−1^ was found in all activated carbon samples, however, the adsorption band continued to decrease in intensity when the activation temperature was increased. This indicates that the amount of large condensed aromatic frame decreases during the activation at a high temperature, corresponding to the consumption of carbon atoms in the graphene layers by the gasification reaction. The stretching vibration of C-H was observed as weak bands at about 2850 to 3000 cm^−1^, mostly in activated carbon samples but was not found in the char sample. The adsorption bands at about 2850 and 2920 cm^−1^ are referred to the symmetric and asymmetric stretching vibration of CH_2_ and CH_3_ groups [36,42]. The intensity of these bands tended to decrease when the activation temperature was increased, indicating the removal of hydrogen content during the activation process [43]. The FTIR results of activated carbon imply that the surface chemistry can be created and destroyed during the activation process, with a high activation temperature being the key parameter that encourages the functional groups to desorb from the surface [44,45]. Due to a high activation temperature and long activation time, most of the functional groups on the surface of the obtained activated carbon tended to decrease significantly as compared to those of the original char.

To substantiate quantitatively the creation and removing of surface groups during the carbonization and physical activation, the Boehm titration technique was used to quantify the change of surface functional groups. The weight fraction of elements in the char and activated carbon samples and the amounts of surface functional groups obtained from the Boehm titration are listed in Table 4. For the char sample, as the carbonization temperature increased, the basic groups appeared to increase. On the other hand, the acidic groups decreased while the carbonization temperature was increased. These results are attributed to the thermal instability of acidic and basic groups with respect to the change in the carbonization temperature; at a higher carbonization temperature most of the acid groups are destroyed, whereas the basic groups are developed [46]. The decrease in the acidic groups is as well confirmed by the decrease in the weight fraction of oxygen contents from the element analysis, as shown in Table 4.

For activated carbon samples, it was found that the total acidic groups increased with the increase in activation temperature, with about 31% increase over the range of temperature from 850 to 1000 °C. The total basic group also showed an increase of 22% when the activation temperature increased from 850 to 950 °C, but tended to drop at the higher temperature of 1000 °C. It is understandable that the acidic surface groups are created and destroyed in parallel along with the development of porous properties during the gasification reactions under CO_2_, with a higher activation temperature creating more oxygen functional groups [47]. As noticed from Table 4, the carboxylic group is the major surface group in bamboo char, while in the prepared activated carbon the phenolic group showed the highest content. The lactonic groups were not detected in all the analyzed samples.

### 2.6. Empirical Correlations for the Formation of the Surface Functional Groups

**Figure 17:**
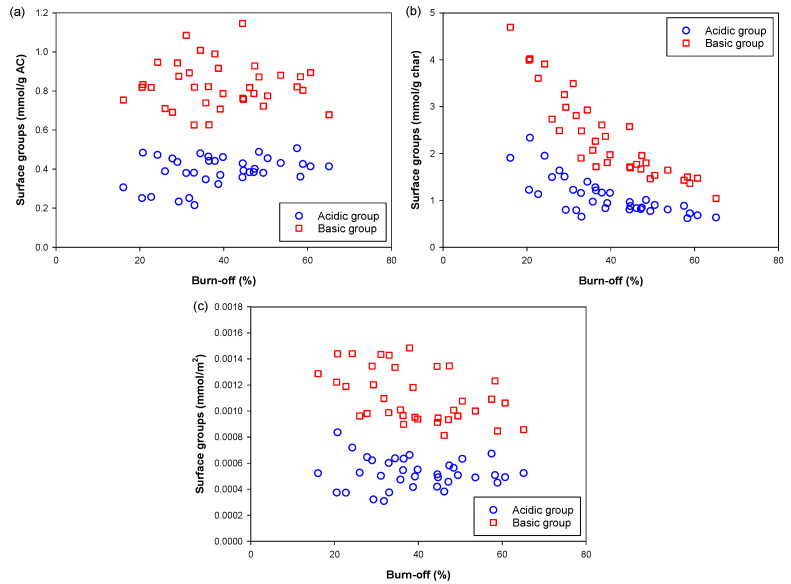
Presentation of surface group concentration in three different units of (**a**) mmol/g carbon, (**b**) mmol/g char and (**c**) mmol/m^2^ of BET surface area, as a function of percentage of char burn-off.

This section is devoted to the development of empirical equations for correlating the number of surface functional groups with the preparation conditions for the production of activated carbon. We commenced by examining the relationship between the concentration of the surface groups and the percentage of char burn-off. Three possible concentration units for the functional groups, mmol/g AC, mmol/g char and mmol/m^2^ of AC surface area, were tried and the plots of functional group concentration versus burn-off are shown in Figure 17. Only the surface concentration in the unit of mmol/g char yielded a clearer relationship. Thus, the data on the amount of surface functional groups in the unit of mmol/g char were used for developing the mathematical correlation between the surface group concentration (acidic and basic groups) and the preparation conditions (carbonization temperature, and activation time and temperature) used in this study.

We started by defining various variables involved. For independent variables, *x*_1_ is given as the carbonization temperature (°C), *x*_2_ is the activation temperature (°C) and *x*_3_ is the activation time (minutes). The dependent variable *Y* represents the response or the concentration of surface functional groups in mmol/g char being obtained from the experimental data. The following relationship can then be written,

(14) $$Y=f\left(x_{1},x_{2},x_{3}\right)+\varepsilon$$

where *ε* is the error or interference that occurs in a system that affects the magnitude of the response.

The experimental data were then fitted with the following second-order polynomial equation of the form,

(15) $$Y=\beta_{0}+\sum_{i=1}^{2}\beta_{i}x_{i}+\sum_{i=1}^{2}\beta_{ii}x_{ii}^{2}+\sum_{i<j}^{1}\sum_{j=1}^{2}\beta_{ij}x_{i}x_{j}+\varepsilon$$

where *Y* is the predicted response, $\beta_{0}$ is a constant, $\beta_{i}$ is a linear term coefficient, $\beta_{ii}$ is a quadratic term coefficient and $\beta_{ij}$ is a cross product term coefficient.

It is more convenient to first put the dependent and independent variables in normalized forms, that is,

(16) $$x_{i,norm}=\frac{x_{i}-x_{\min}}{x_{diff}}$$

(17) $$Y_{i,norm}=\frac{Y_{i}-Y_{\min}}{Y_{diff}}$$

where $x_{i,norm}$ and $Y_{i,norm}$ are the normalized values of the independent variable, $x_{i}$ and $Y_{i}$ dependent variables, $x_{\min}$ and $Y_{\min}$ are the minimum values of $x_{i}$ and $Y_{i}$, and $x_{diff}$ and $Y_{diff}$ are the difference between the maximum and minimum values of $x_{i}$ and $Y_{i}$, respectively. Equation (15) was used to fit the normalized values of *Y* and *x* from experiments and a non-linear regression was applied to obtain the values of constants in the equation by minimizing the overall error. The final equations in normalized forms with the fitted constants were first derived from data fitting and then they were converted back to the normal forms of the dependent and independent variables. The final derived equations are the following.

(18) $$\begin{array}{l} Y_{acid}=26.3872+4.1162\times 10^{-2}T_{C}-6.6906\times 10^{-2}T_{Act}-5.3928\times 10^{-2}t_{Act}-1.0685\times 10^{-5}T_{C}T_{Act}+1.5745\times 10^{-5}T_{C}t_{Act}\\ \;+2.5878\times 10^{-5}T_{Act}t_{Act}-3.1749\times 10^{-5}T_{C}^{2}+3.5153\times 10^{-5}T_{Act}^{2}+1.0291\times 10^{-4}t_{Act} \end{array}$$

(19) $$\begin{array}{l} Y_{base}=-12.7457+9.9663\times 10^{-2}T_{C}-1.6788\times 10^{-2}T_{Act}+3.7510\times 10^{-2}t_{Act}-4.1479\times 10^{-5}T_{C}T_{Act}-1.0268\times 10^{-4}T_{C}t_{Act}\\ \;+2.3321\times 10^{-5}T_{Act}t_{Act}-4.6442\times 10^{-5}T_{C}^{2}+1.4370\times 10^{-5}T_{Act}^{2}-1.3059\times 10^{-4}t_{Act}^{2} \end{array}$$

where $Y_{acid}$ and $Y_{basic}$ are the amounts of acid and basic surface groups in mmol/g char, respectively. *T_C_* is the carbonization temperature (400 to 600 °C), *T_Act_* is the activation temperature (850 to 1000 °C), and *t_Act_* is the activation time (60 to 120 min). The R^2^ of Equations (18) and (19) are 0.821 and 0.9254, with standard error of 0.1203 and 0.0784, respectively.

**Figure 18:**
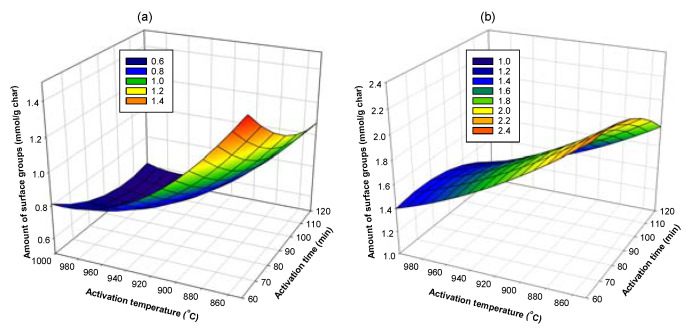
Surface response curves showing the effects of activation temperature and activation time on the amount of surface groups on activated carbon derived from char C400-90, (**a**) acidic groups and (**b**) basic groups.

**Figure 19:**
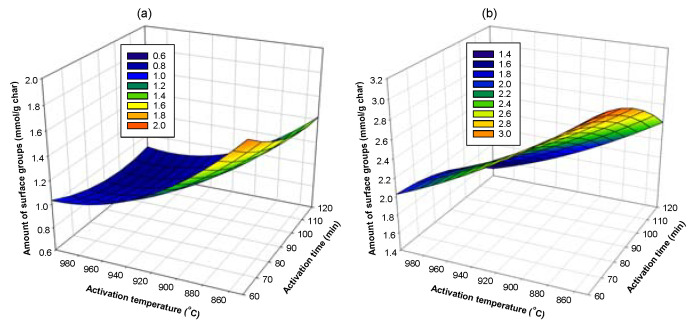
Surface response curves showing the effects of activation temperature and activation time on the amount of surface groups on activated carbon derived from char C500-90, (**a**) acidic groups and (**b**) basic groups.

**Figure 20:**
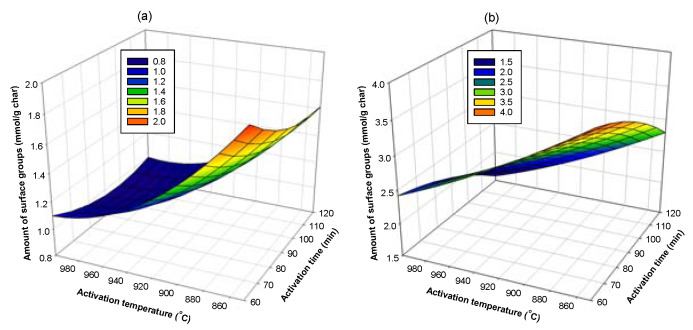
Surface response curves showing the effects of activation temperature and activation time on the amount of surface groups on activated carbon derived from char C600-90, (**a**) acidic groups and (**b**) basic groups.

Next, Equations (18) and (19) were used to determine the optimum conditions that give the highest amounts of acid and basic surface groups on activated carbon by plotting a surface response of the dependent variable (amount of surface groups) as a function of the independent variables (preparation conditions), as typically shown in Figure 18, Figure 19 and Figure 20. The optimum conditions for creating the acidic surface groups were found to be 520 °C and 90 min for carbonization conditions and activation temperature of 850 °C with 60 min of activation time. For the formation of maximum basic groups, the optimum conditions are 600 °C and 90 min for carbonization conditions and the activation conditions of 850 °C and 60 min. These conditions are reasonable, considering the fact that a too high activation temperature and long activation time used during the activation process could destroy the number of surface groups formed in the activated carbon.

## 3. Materials and Methods

### 3.1. Raw Material

Bamboo (*Bambusa Bambos*) was used as the starting raw material in this study. The as-received bamboo was chopped and cut into square pieces and sieved to obtain an average particle size of 2.03 mm (8 × 12 mesh). Next, the raw material was rinsed thoroughly with DI water to eliminate all dirt and contaminants, and then it was dried at 110 °C for 24 h in an electric oven to remove excess moisture. The obtained sample was kept in a desiccator for further analysis and the preparation of char.

### 3.2. Raw Material Characterization

The prepared bamboo was analyzed by a thermogravimetric analyzer (TGA/DSC-1 Star System, Mettler-Toledo, Greifensee, Switzerland) for the proximate analysis [48] to determine moisture, volatile matter, fixed carbon, and ash content [49]. The ultimate analysis was performed by a CHN analyzer (CHN 628, Leco Corporation, St. Joseph, MI, USA). The results obtained were weight percent of carbon, hydrogen, and nitrogen. The weight percent of oxygen was determined by mass balance, that is, %O = 100 − [%C + %H + %N].

### 3.3. Char Preparation

A dried bamboo sample weighing about 30 g was loaded into an alumina ceramic boat and placed in a horizontal ceramic tube furnace (CTF 12/75/700, Carbolite, Staffordshire, UK) of diameter 75 mm with N_2_ gas (99.995% of purity, Thai Special Gas, Rayong, Thailand) flowing through at the rate of 100 cm^3^/min. The carbonization was programmed by heating the furnace from the ambient temperature to 400 °C at a heating rate of 10 °C/min and then it was held at this temperature for 90 min. Then, the furnace was switched off and the product was cooled down inside the horizontal tube furnace to the ambient temperature under a constant flow of N_2_. Finally, the char produced was removed from the furnace and kept in a desiccator for further activation in CO_2_. The carbonization temperatures studied were 400, 500 and 600 °C and the carbonization time was kept constant at 90 min for all runs. The weights of the dried bamboo and that of the derived char were recorded to determine the yield of the char product.

### 3.4. Activated Carbon Production

The activation of carbonized char was carried out in the same tube furnace that was set in a vertical position. A quartz tube was inserted into the furnace to use as a reactor for char gasification. A total of 25 g of carbonized char were loaded into the quartz tube. The top and bottom of the furnace were insulated with ceramic fiber blankets to prevent heat losses to the surrounding area. The heating temperature was programmed to increase from the ambient temperature to the desired activation temperature at a heating rate of 10 °C/min under the flow of N_2_ at 100 cm^3^/min. When the desired activation temperature was reached, N_2_ was stopped and CO_2_ (99.995% of purity, Thai Special Gas, Rayong, Thailand) was immediately admitted into the quartz tube reactor at a constant flow rate of 100 cm^3^/min. After the char was activated with CO_2_ for the required period of time, the CO_2_ valve was closed and N_2_ was then allowed to flow into the quartz tube at the rate of 100cm^3^/min. The furnace was then switched off and the product was cooled down in the furnace to the ambient temperature under a constant flow of N_2_. The obtained activated carbon product was kept in a desiccator for further characterization. The activation conditions studied were 850, 900, 950 and 1000 °C and the holding times were 60, 90 and 120 min for each activation temperature. As an example, the prepared activated carbon was designated as C400-90 AC850-60 to denote that this activated carbon product was derived from the activation temperature of 850 °C for 60 min and using the char prepared at 400 °C for 90 min.

The degree of char burn-off or char conversion in weight% was computed from the following equation,

(20) $$Burn-off\left(\%\right)=\frac{w_{char}-w_{AC}}{w_{char}}\times 100$$

where $w_{char}$ and $w_{AC}$ are the weights of char before activation and of the obtained activated carbon, respectively.

### 3.5. Porous Properties of Activated Carbon Products

The porous properties of the derived activated carbon were determined from the adsorption/desorption isotherm data of N_2_ (99.9999% of purity, Linde Thailand, Rayong, Thailand) at −196 °C, measured with a high-performance adsorption analyzer (ASAP 2010, Micromeritics, Norcross, GA, USA). The sample was first loaded into a sample tube and degassed at 300 °C under a vacuum pressure of below 10 µmHg for 12 h. Then, the sample tube was transferred to the analysis port to perform the adsorption/desorption of N_2_ at −196 °C for a pressure range of up to 1 atm. The Brunauer-Emmett-Teller (BET) equation [50] was applied to calculate the specific surface area (*S_BET_*) using the N_2_ adsorption isotherm data for the relative pressures (P/P_0_) of 0.05 to 0.25 [31]. The total pore volume (*V_tot_*) was determined from the volume of N_2_ adsorbed at a relative pressure of 0.98 and converted to the volume of N_2_ in a liquid state at −196 °C, assuming that the adsorbed N_2_ is in the liquid-like state at the temperature of adsorption. The micropore volume (*V_mic_*) for pore sizes smaller than 2.0 nm was determined from the Dubinin-Radushkevich equation [51], while the volume of mesopores (*V_mes_*) was derived from the difference between the total pore volume (*V_tot_*) and the micropore volume. The average pore diameter was also determined using the relation 4*V_tot_*/*S_BET_*, where *V_tot_* is the total pore volume and *S_BET_* is the BET surface area. The pore size distribution (PSD) of the prepared activated carbon was computed based on the Grand Canonical Monte Carlo (GCMC) simulation technique of which a brief theory is provided in Section 3.8.

### 3.6. Boehm’s Titration

Boehm titration [52,53] is one of the most widely used methods to determine the quantity of oxygen functional groups on the surface of activated carbon. Based on this method, it is assumed that the acidic groups on the surface of activated carbon are neutralized with bases of varying strength: (1) carboxylic group in a NaHCO_3_ solution, (2) carboxylic and lactonic groups in a Na_2_CO_3_ solution and (3) a combination of carboxylic, lactonic and phenolic groups in a NaOH solution. To determine the basic group contents on the activated carbon surface, HCl solution was employed to neutralize the basic groups. The titration procedures were mentioned elsewhere [54]. In brief, the following steps were employed: (1) About 4 g of activated carbon were crushed in a mortar and sieved with a 20-mesh screen (850 µm). The under-size particles (−850 µm) weighing about 1 g were collected and loaded into each of the four conical flasks (the exact weight of carbon in each conical flask was recorded). (2) Each of the first three conical flasks was mixed with 50 cm^3^ of 0.1 M solutions of HCl, NaOH and NaHCO_3_, respectively. The last conical flask was mixed with 50 cm^3^ of 0.05 M solution of Na_2_CO_3_. (3) All flasks were sealed and shaken at 150 rpm for 24 h at the ambient temperature and after that the mixture was filtered. A total of 10 cm^3^ of the respective filtered solutions of NaOH, Na_2_CO_3_ and NaHCO_3_ were titrated with 0.1 M HCl solution, while the filtered solution of HCl was titrated with 0.1 M NaOH solution. (4) The volumes of titrants of the various bases and acids were used to calculate the number of various oxygen functional groups on the surface of the activated carbon.

### 3.7. FTIR Spectra

Fourier Transform Infrared (FTIR) spectroscopy (Vertex 70 FT-IR, Bruker, Billerica, MA, USA) was employed to identify the functional groups present on the surface of the prepared char and activated carbon. The infrared spectra from a light source were ascertained over the wave number from 4000 to 400 cm^−1^. For each sample run, 64 scans were performed with a resolution value of 4 cm^−1^.

### 3.8. Pore Size Distribution (PSD) by Grand Canonical Monte Carlo (GCMC) Simulation

A brief description of GCMC simulation for determining the pore size distribution of activated carbon is presented in this section. In this work, Monte Carlo (MC) simulation [55] in the grand canonical (GC) ensemble [56] was employed to obtain the kernel of reference adsorption isotherms. Pores were modelled as perfect slit pores with pore widths, *H*, ranging from 0.65 to 4.0 nm. The distance in the *x*- and *y*-directions was 6.0 × 6.0 nm^2^, while the pore width, *H*, was in the *z*-direction. Periodic boundary conditions (PBC) were applied in the *x*- and *y*-directions and the cut-off radius was chosen to be 5 times the collision diameter of the fluid. The pore wall was modelled using the homogeneous graphite surface proposed by Steele [57] with the following values of the pertinent parameters; reduced well depth of solids $\varepsilon_{ss}/k_{B}=28K$, the interspacing between graphene layers, Δ=0.3354 nm, the collision diameter of carbon atoms, σ=0.34 nm and the density of the carbon surface, $\rho_{s}=38.2{\;\mathrm{nm}}^{-2}$. The interaction energy between the solid and the fluid $\left(\varphi_{sf}\right)$ was described using the following Steele 10-4-3 equation [57]:

(21) $$\varphi_{sf}\left(z\right)=2\pi \rho_{s}\varepsilon_{sf}\sigma_{sf}^{2}\left\{\frac{2}{5}{\left(\frac{\sigma_{sf}}{z}\right)}^{10}-{\left(\frac{\sigma_{sf}}{z}\right)}^{4}-\left[\frac{\sigma_{sf}^{4}}{3\Delta {\left(z+0.61\Delta \right)}^{3}}\right]\right\}$$

where *z* is the distance from the Lennard-Jones (LJ) site to the centers of carbon atoms in the outermost layer of the graphene sheet. The $\varepsilon_{sf}$ is the cross-well depth and $\sigma_{sf}$ is the cross-collision diameter of the solid-fluid interaction energy, they were calculated using Equations (23) and (24).

The pair-wise fluid and fluid $\left(\varphi_{ff}\right)$ interaction energy between molecules *i* and *j* was calculated by the Lennard-Jones (LJ) 12-6 equation as shown in Equation (22)

(22) $$\varphi_{ff}\left(r\right)=\sum_{a=1}^{A}\sum_{b=1}^{B}4\varepsilon_{i,j}^{a,b}\left[{\left(\frac{\sigma_{i,j}^{a,b}}{r_{i,j}^{a,b}}\right)}^{12}-{\left(\frac{\sigma_{i,j}^{a,b}}{r_{i,j}^{a,b}}\right)}^{6}\right]$$

where *A* and *B* are the number of LJ sites on molecule *i* and *j*, respectively. $r_{i,j}^{a,b}$ is the separation distance between the LJ site *a* of molecule *i* and the LJ site *b* of molecule *j*, while $\varepsilon_{i,j}^{a,b}$ is the cross-well depth and $\sigma_{i,j}^{a,b}$ is the cross-collision diameter.

Nitrogen was modelled as a single LJ molecule as proposed by Ravikovitch et al. [58]. The reduced well depth of the fluid was $\varepsilon_{ff}/k_{B}=101.5K$, and a collision diameter was $\sigma_{ff}=0.3615\;\mathrm{nm}$.

The cross-well depth $\left(\varepsilon_{sf}/k_{B}\right)$ and cross-collision diameter $\left(\sigma_{sf}\right)$ between solid-fluid were determined by the Lorentz-Berthelot (LB) mixing rule as follows:

(23) $$\sigma_{sf}=\left(\sigma_{ss}+\sigma_{ff}\right)/2$$

(24) $$\varepsilon_{sf}={\left(\varepsilon_{ss}\varepsilon_{ff}\right)}^{1/2}$$

A family of nitrogen adsorption isotherms at −196 °C were determined using GCMC simulation and 10^5^ cycles were executed in the equilibration stage, and the same number in the sampling stage. In each cycle, there were 1000 trial moves of local displacement, insertion, and deletion with equal probabilities. In the equilibration stage, the maximum displacement length was initially set as one half of the largest dimension of the simulation box and was adjusted at the end of each cycle to give an acceptance ratio of 20% [59]. The chemical potential for a given temperature and pressure, used as input in GCMC, was calculated using the equation of state (EoS) of Johnson et al. [60]. The adsorbed density was expressed as the absolute adsorbed density in slit pore per unit accessible volume [61] as a function of pressure.

The experimental N_2_ isotherms at −196 °C of activated carbon and the theoretical isotherms from GCMC are then paired and optimized using Solver in Microsoft Excel to minimize the sum square error between the experimental and simulated isotherms. The pore size distribution (PSD) of activated carbon can then be derived as the relationship between the differential pore volume per unit pore width and the average pore width.

## 4. Conclusions

Bamboo was used as a precursor for the synthesis of activated carbon by the two-step physical activation with carbon dioxide and the effects of carbonization temperature (400 to 600 °C), activation temperature (850 to 1000 °C) and activation time (60 to 120 min) on the pore development and the formation of oxygen functional groups were investigated. Increasing activation conditions promoted the porous properties of produced activated carbons, but at a relatively high degree of char burn-off the surface area and micropore volume decreased with the corresponding increase in mesopore volume. Under the preparation conditions investigated, the activated carbon prepared from bamboo contained mostly micropores (>85% of total pore volume). The maximum surface area of 907 m^2^/g and total pore volume of 0.446 cm^3^/g were obtained at carbonization temperature of 400 °C and 90 min, activation temperature of 900 °C and 120 min. Under the same activation conditions, activated carbon prepared from the char carbonized at the lowest temperature of 400 °C gave the highest porous properties, presumably because of having a larger number of active sites for the CO_2_ gasification. The results of pore size distribution of produced activated carbons indicate that most pores were developed in the micropore size range of 0.65 to 1.4 nm. Empirical correlations were proposed to predict all the important porous properties of the bamboo-based activated carbons as functions of carbonization temperature and activation conditions. It is obvious that the creation of surface functional groups by the oxidation of CO_2_ takes place in parallel with the development of carbon pores. It was found that the amounts of acidic surface functional groups in the char samples decreased with the increase in carbonization temperature, while the opposite effect occurred for the contents of the basic groups. When the activation temperature increased, both the contents of acid and basic groups tended to increase. Empirical equations were developed to estimate the amounts of the total acid and basic surface groups in bamboo-activated carbons in terms of carbonization temperature and activation conditions. The knowledge of the concentration of oxygen functional groups of activated carbon as a function of preparation conditions would be beneficial to the selection of experimental conditions for oxidizing activated carbon, by air or an acid, with the purpose being to effectively increase the contents of surface functional groups.

## Figures and Tables

**Table 1:** Proximate and ultimate analyses of bamboo used in this work.

**Analysis Method**	
**Proximate analysis**	**wt%**
Moisture	6.97
Volatile compounds	73.02
Fixed carbon	19.91
Ash	0.10
**Ultimate analysis**	**wt%**
C	45.33
H	6.13
N	0.40
O (by difference)	48.14

**Table 2:** BET surface area of chars derived from CO_2_ adsorption at 0 °C for chars prepared at different carbonization temperature of 400, 500 and 600 °C.

Char Sample	BET Surface Area, *S*_0_, (m^2^/g)
C400-90	144
C500-90	178
C600-90	219

**Table 3:** Distribution of pore volume for various pore sizes in activated carbon prepared under different preparation conditions.

Run no.	Sample	Pore Volume for Pore Width (cm^3^/g)					*V_tot_* (cm^3^/g)
		0.0–0.65 nm	0.65–1.4 nm	1.4–2.0 nm	2.0–3.0 nm	3.0–4.0 nm	
	from C400-90						
1	AC850-60	0.000	0.2344	0.0072	0.000	0.0006	0.2539
2	AC900-60	0.000	0.2757	0.0122	0.000	0.0171	0.3181
3	AC950-60	0.000	0.3155	0.0012	0.000	0.0332	0.3586
4	AC1000-60	0.000	0.2934	0.0074	0.000	0.0274	0.3373
5	AC850-90	0.000	0.2700	0.0001	0.000	0.000	0.2851
6	AC900-90	0.000	0.3206	0.0008	0.000	0.0205	0.3616
7	AC950-90	0.000	0.3479	0.004	0.000	0.0427	0.3986
8	AC1000-90	0.000	0.3254	0.015	0.000	0.0483	0.3988
9	AC850-120	0.000	0.2834	0.0001	0.000	0.0270	0.3145
10	AC900-120	0.000	0.3577	0.0176	0.000	0.0768	0.4458
11	AC950-120	0.000	0.3292	0.0442	0.000	0.0762	0.4546
12	AC1000-120	0.000	0.2484	0.0507	0.000	0.0684	0.3845
	from C500-90						
13	AC850-60	0.000	0.2095	0.0001	0.000	0.000	0.2337
14	AC900-60	0.000	0.2535	0.0003	0.000	0.0133	0.294
15	AC950-60	0.000	0.2832	0.0005	0.000	0.028	0.3302
16	AC1000-60	0.000	0.2551	0.0011	0.000	0.0275	0.2964
17	AC850-90	0.000	0.2425	0.0004	0.000	0.0079	0.2722
18	AC900-90	0.000	0.2807	0.0024	0.000	0.0236	0.3279
19	AC950-90	0.000	0.3183	0.0072	0.000	0.0509	0.3936
20	AC1000-90	0.000	0.2624	0.0138	0.000	0.0364	0.3284
21	AC850-120	0.000	0.2682	0.0017	0.000	0.0032	0.2885
22	AC900-120	0.000	0.2939	0.029	0.000	0.0202	0.3646
23	AC950-120	0.000	0.2795	0.0498	0.000	0.0383	0.3823
24	AC1000-120	0.000	0.2193	0.0582	0.000	0.0457	0.3353
	from C600-90						
25	AC850-60	0.000	0.2084	0.0011	0.000	0.000	0.2328
26	AC900-60	0.000	0.2466	0.0031	0.000	0.0132	0.2863
27	AC950-60	0.000	0.2639	0.0106	0.000	0.0205	0.3164
28	AC1000-60	0.000	0.1997	0.0168	0.000	0.013	0.2501
29	AC850-90	0.000	0.2377	0.0006	0.000	0.000	0.2656
30	AC900-90	0.000	0.2750	0.0174	0.000	0.0213	0.3492
31	AC950-90	0.000	0.2537	0.027	0.000	0.0272	0.3404
32	AC1000-90	0.000	0.2135	0.0381	0.000	0.0407	0.3178
33	AC850-120	0.000	0.2732	0.0017	0.000	0.0037	0.3034
34	AC900-120	0.000	0.2691	0.0349	0.000	0.0349	0.3776
35	AC950-120	0.000	0.249	0.0665	0.000	0.055	0.4054
36	AC1000-120	0.000	0.208	0.0703	0.000	0.0536	0.3584

**Table 4:** Typical elemental analysis and surface chemistry of prepared char and the prepared activated carbon. All activated carbon samples were derived from char carbonized at 400 °C for 90 min.

Sample	Elemental Analysis (wt%)				Amount of Surface Functional Groups (mmol/g)				
	C	H	N	O	Carboxylic	Lactonic	Phenolic	Total Acidic	Total Basic
C400-90	56.0	3.8	0.5	39.7	0.37	0.00	0.40	0.77	0.21
C500-90	50.4	3.1	0.4	46.1	0.44	0.00	0.11	0.55	0.38
C600-90	52.5	2.4	0.4	44.6	0.32	0.00	0.00	0.32	0.44
AC850-120	60.0	1.8	0.5	37.7	0.04	0.00	0.35	0.39	0.71
AC900-120	60.0	1.7	0.6	37.7	0.01	0.00	0.45	0.46	0.82
AC950-120	57.9	1.5	0.6	40.0	0.06	0.00	0.43	0.49	0.87
AC1000-120	53.2	1.6	0.4	44.8	0.08	0.00	0.43	0.51	0.82

## Data Availability

The data presented in this study will be available upon request.

## Sample Availability

Samples of the compounds are not available from the authors.

## Appendix A

**Table A1:** Porous properties of the prepared activated carbons.

Run No.	Sample	*S_BET_* (m^2^/g)	*V_mic_* (cm^3^/g)	*V_mes_* (cm^3^/g)	*V_tot_* (cm^3^/g)	*D_avg_* (nm)	Burn-Off (%)
	from C400-90						
1	AC850-60	584	0.2432 (95.8%)	0.0107 (4.2%)	0.2539	1.74	32.9
2	AC900-60	666	0.2879 (90.5%)	0.0302 (9.5%)	0.3181	1.91	39.2
3	AC950-60	728	0.3145 (87.7%)	0.0441 (12.3%)	0.3586	1.93	44.7
4	AC1000-60	693	0.2980 (88.3%)	0.0393 (11.7%)	0.3373	1.95	49.4
5	AC850-90	621	0.2691 (94.4%)	0.0160 (5.6%)	0.2851	1.84	36.5
6	AC900-90	753	0.3265 (90.3%)	0.0351 (9.7%)	0.3616	1.92	44.5
7	AC950-90	810	0.3498 (87.8%)	0.0488 (12.2%)	0.3986	1.97	53.6
8	AC1000-90	787	0.3377 (84.7%)	0.0611 (15.3%)	0.3988	2.02	60.7
9	AC850-120	654	0.2830 (90.0%)	0.0315 (10.0%)	0.3145	1.92	35.7
10	AC900-120	907	0.3943 (88.4%)	0.0515 (11.6%)	0.4458	1.97	46.2
11	AC950-120	884	0.3811 (83.8%)	0.0735 (16.2%)	0.4546	2.06	58.9
12	AC1000-120	749	0.3182 (82.8%)	0.0663 (17.2%)	0.3845	2.05	65.1
	from C500-90						
13	AC850-60	513	0.2221 (95.0%)	0.0116 (5.0%)	0.2337	1.82	20.7
14	AC900-60	628	0.2716 (92.4%)	0.0224 (7.6%)	0.294	1.87	29.0
15	AC950-60	685	0.2956 (89.5%)	0.0346 (10.5%)	0.3302	1.93	34.4
16	AC1000-60	614	0.2644 (89.2%)	0.0320 (10.8%)	0.2964	1.93	37.9
17	AC850-90	585	0.2532 (93.0%)	0.0190 (7.0%)	0.2722	1.86	24.2
18	AC900-90	684	0.2956 (90.1%)	0.0323 (9.9%)	0.3279	1.92	31.1
19	AC950-90	786	0.3405 (86.5%)	0.0531 (13.5%)	0.3936	2.00	44.5
20	AC1000-90	666	0.2870 (87.4%)	0.0414 (12.6%)	0.3284	1.97	50.5
21	AC850-120	627	0.2716 (94.1%)	0.0169 (5.9%)	0.2885	1.84	27.8
22	AC900-120	764	0.3299 (90.5%)	0.0347 (9.5%)	0.3646	1.90	39.8
23	AC950-120	782	0.3352 (87.7%)	0.0471 (12.3%)	0.3823	1.96	47.2
24	AC1000-120	665	0.2848 (84.9%)	0.0505 (15.1%)	0.3353	2.02	58.3
	from C600-90						
25	AC850-60	518	0.2243 (96.3%)	0.0085 (3.7%)	0.2328	1.80	16.1
26	AC900-60	614	0.2662 (93.0%)	0.0201 (7.0%)	0.2863	1.86	22.7
27	AC950-60	660	0.2859 (90.4%)	0.0305 (9.6%)	0.3164	1.92	29.3
28	AC1000-60	529	0.2265 (90.6%)	0.0236 (9.4%)	0.2501	1.89	33.0
29	AC850-90	594	0.2577 (97.0%)	0.0079 (3.0%)	0.2656	1.79	20.5
30	AC900-90	734	0.3185 (91.2%)	0.0307 (8.8%)	0.3492	1.90	31.8
31	AC950-90	709	0.3053 (89.7%)	0.0351 (10.3%)	0.3404	1.92	38.7
32	AC1000-90	642	0.2748 (86.5%)	0.0430 (13.5%)	0.3178	1.98	47.4
33	AC850-120	660	0.2854 (94.1%)	0.0180 (5.9%)	0.3034	1.84	26.0
34	AC900-120	776	0.3359 (89.0%)	0.0417 (11.0%)	0.3776	1.95	36.3
35	AC950-120	803	0.3461 (85.4%)	0.0593 (14.6%)	0.4054	2.02	48.4
36	AC1000-120	708	0.3018 (84.2%)	0.0566 (15.8%)	0.3584	2.02	57.5
